# Migration of Vasoconstriction in Reversible Cerebral Vasoconstriction Syndrome

**DOI:** 10.31662/jmaj.2022-0169

**Published:** 2022-11-30

**Authors:** Tetsuya Hashimoto, Ayano Matsuyoshi, Wataru Shiraishi

**Affiliations:** 1 Department of Neurology, Kokura Memorial Hospital, Kitakyushu, Japan

**Keywords:** Magnetic resonance imaging, reversible cerebral vasoconstriction syndrome, thunderclap headache

A 49-year-old woman developed thunderclap headaches triggered by straining during defecation. Magnetic resonance imaging (MRI) on day 3 after the onset revealed somewhat poor delineation of the bilateral distal posterior cerebral arteries without cerebral parenchymal lesions, which suggested possible vasoconstriction (Figure 1A and 1B). There were no intra-arterial signals on fluid-attenuated inversion recovery images and thereafter headaches during defecation remained. On day 17, although the visualization of the posterior cerebral arteries was improved, the basilar artery showed segmental constriction (Figure 1C). Basi-parallel anatomical scanning revealed segmental constriction of the external vessel wall of the basilar artery (Figure 1D). Reversible cerebral vasoconstriction syndrome (RCVS) was diagnosed and verapamil was administered. The patient’s headaches gradually disappeared and vasoconstriction was resolved on MRI on day 31.

**Figure 1.**
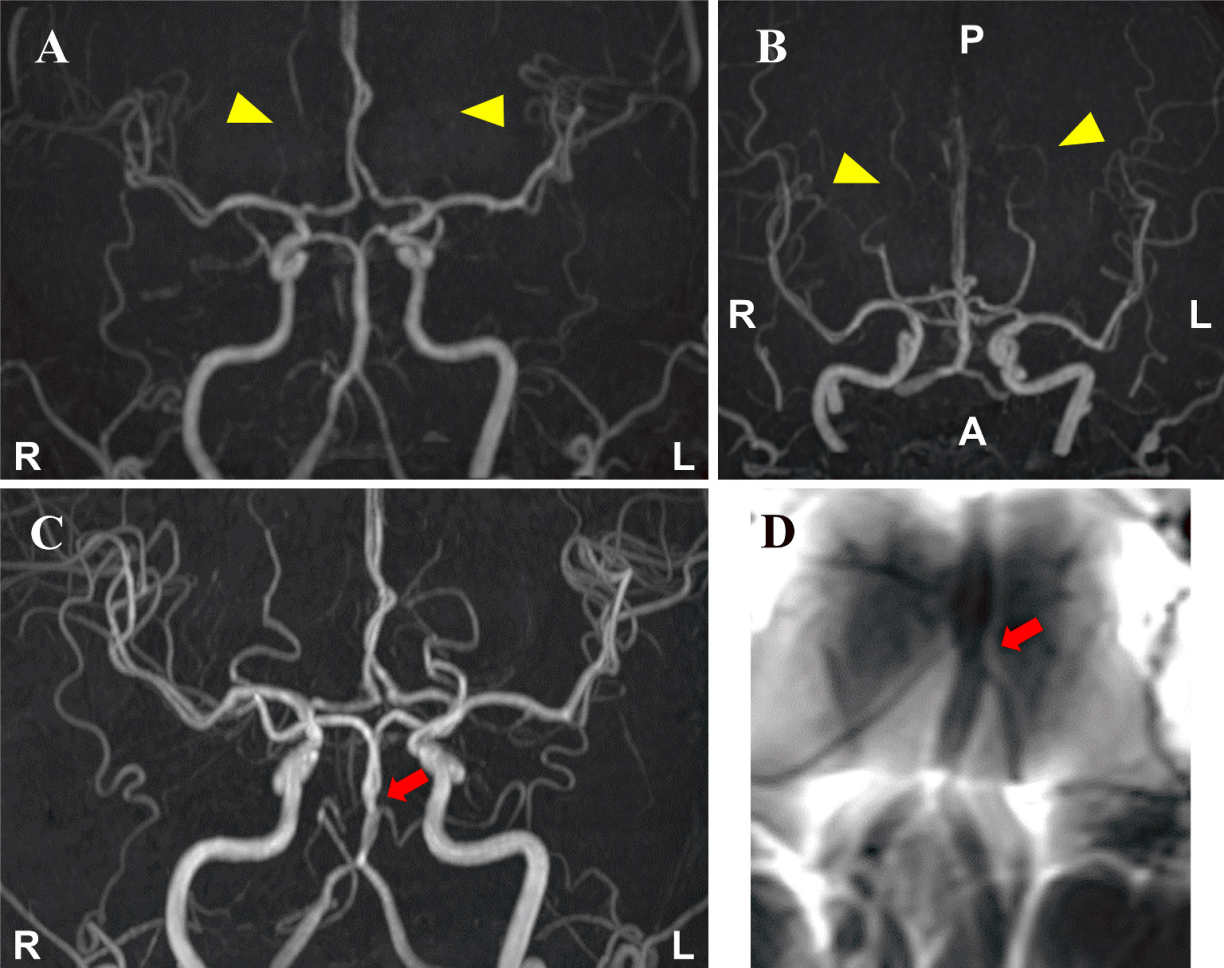
Magnetic resonance angiography (MRA) on day 3 after the onset revealed somewhat poor delineation of the bilateral distal posterior cerebral arteries (arrowheads), which suggested possible vasoconstriction (A, B). MRA on day 17 showed segmental constriction of the basilar artery (arrow) (C). Basi-parallel anatomical scanning on the same day revealed segmental constriction of the external vessel wall of the basilar artery (arrow) (D).

In RCVS, vasoconstriction can be difficult to detect in very distal branches, which can make its diagnosis challenging ^(1)^. Vasoconstriction has been reported to start at distal vessels and migrate more proximally to larger vessels ^(2)^. Therefore, serial MRI examinations are important for diagnosing RCVS.

## Article Information

### Conflicts of Interest

None

### Author Contributions

All authors contributed to patient care. Tetsuya Hashimoto wrote the manuscript and the other authors revised it.

### Approval by Institutional Review Board (IRB)

This study did not require IRB approval.

### Informed Consent

Written informed consent was obtained from the patient to publish.
